# Effect of enteral nutrition support combined with prone position mechanical ventilation on respiratory function, nutritional status, and inflammatory response in patients with severe pneumonia

**DOI:** 10.3389/fphys.2026.1739744

**Published:** 2026-02-23

**Authors:** Li Xu, Ling Xie, Huijuan Wang

**Affiliations:** 1 Department of Clinical Nutrition, First People’s Hospital of Linping District, Hangzhou, Zhejiang, China; 2 Department of Critical Medicine, First People’s Hospital of Linping District, Hangzhou, Zhejiang, China

**Keywords:** adverse events, enteral nutrition support, inflammation, nutritional status, prone position mechanical ventilation, severe pneumonia

## Abstract

**Objective:**

This study aims to investigate the efficacy of enteral nutrition support combined with prone position mechanical ventilation in patients with severe pneumonia.

**Methods:**

This retrospective cohort study included 55 patients with severe pneumonia, who were allocated to a control group (n = 35) receiving conventional mechanical ventilation combined with early enteral nutrition support, and an observation group (n = 20) receiving prone position mechanical ventilation combined with early enteral nutrition support. The intervention lasted for 1 week. Changes in blood gas indicators were compared before and after the intervention. Improvement in nutritional status and inflammatory indicators, including serum prealbumin (PAB), albumin (ALB), haemoglobin (HGB) and C-reactive protein (CRP), and procalcitonin (PCT), were assessed. The incidence of adverse events during the intervention was compared between groups. This study was approved by the Ethics Review Committee of our hospital, and written informed consent was obtained from all participants.

**Results:**

After the intervention, both groups showed increased PaO_2_, SpO_2_, and PaO_2_/FiO_2_ levels and decreased PaCO_2_ levels, with more pronounced improvement observed in the observation group. Nutritional indicators (PAB, ALB, and HGB) improved in the observation group. CRP and PCT levels were reduced in both groups, with the observation group demonstrating lower levels. The observation group showed a lower incidence of adverse events than the control group (15.00% vs. 42.86%).

**Conclusion:**

Enteral nutrition support combined with prone position mechanical ventilation reduces the incidence of adverse events, improves respiratory function and nutritional status, and alleviates inflammatory response in patients with severe pneumonia.

## Introduction

Severe pneumonia remains a critical condition with high morbidity and mortality worldwide, posing a substantial burden to healthcare systems (Cilloniz et al., 2021). Characterized by acute respiratory distress, systemic inflammatory responses, and organ dysfunction, severe pneumonia requires timely and effective interventions to prevent disease progression and improve clinical outcomes (Cilloniz et al., 2022). Mechanical ventilation is a cornerstone in managing respiratory failure in these patients, as it helps maintain adequate oxygenation and ventilation (Grotberg et al., 2023). Specifically, mechanical ventilation involves the use of ventilatory support to assist breathing by delivering oxygen to the lungs and facilitating the elimination of carbon dioxide (Walter, 2021).

In recent years, prone position mechanical ventilation has received increasing attention in the treatment of acute respiratory distress syndrome (ARDS) and severe pneumonia (Qin et al., 2024; Wong et al., 2022). By redistributing transpulmonary pressure and lung stress, prone positioning facilitates alveolar recruitment, improves ventilation–perfusion matching, and enhances oxygenation (Papazian et al., 2022). This technique also reduces dorsal lung collapse and limits overinflation of ventral lung regions, thereby contributing to a more homogenous ventilation pattern (Fayed et al., 2023). Consequently, prone position mechanical ventilation has been increasingly recognized as an effective approach for improving respiratory function in critically ill patients. Evidence has shown that compared with the supine position, prone position ventilation improves oxygenation indices and shortens the duration of mechanical ventilation in patients undergoing cardiopulmonary bypass surgery without causing significant hemodynamic instability (Li et al., 2024). However, in patients of severe pneumonia, the underlying pathophysiology is more complex, and the therapeutic effects of prone positioning warrant further investigation.

In addition to respiratory support, nutritional support has become an essential component of comprehensive care for patients with severe pneumonia (Bradbury et al., 2023). Malnutrition and metabolic disturbances are common in critically ill patients and may exacerbate inflammatory responses, impair immune function, and delay recovery. Early enteral nutrition support has been shown to reduce complications, shorten hospital stay, and improve clinical outcomes at discharge (Allen and Hoffman, 2019). Compared to parenteral nutrition, enteral feeding helps preserve gut mucosal integrity and supports immune homeostasis, and is therefore recommended as preferred nutritional support strategy in critically ill patients (Abunnaja et al., 2013).

Despite the individual benefits of prone position ventilation and enteral nutrition support (Walter and Ricard, 2023; Jordan and Moore, 2020), evidence regarding their combined application in severe pneumonia remains limited. The integration of these two interventions may offer a more comprehensive approach by simultaneously addressing respiratory dysfunction and nutritional deterioration, while potentially alleviating systemic inflammatory responses. Therefore, this study aimed to evaluate the combined effects of prone position mechanical ventilation and enteral nutrition support in patients with severe pneumonia. By assessing changes in respiratory parameters, nutritional indices, inflammatory markers, and the incidence of adverse events, this study seeks to provide clinical evidence for the feasibility and effectiveness of this combined therapeutic strategy.

## Materials and methods

### Ethics statement

The study was approved by the Institutional Review Board of First People’s Hospital of Linping District and written informed consent was obtained from all patients.

### Study design and study population

This study was designed as a retrospective cohort study. A total of 55 patients with severe pneumonia admitted to the First People’s Hospital of Linping District from August 2019 to December 2023 were included. Patients were assigned to a control group (n = 35) receiving conventional mechanical ventilation combined with early enteral nutrition support, or an observation group (n = 20) receiving prone position mechanical ventilation combined with early enteral nutrition support.

The inclusion criteria were as follows: ① Patients meeting the diagnostic criteria for severe pneumonia, defined by laboratory findings (blood urea nitrogen >20 mg/dL, white blood cell count <4,000 cells/mm^3^, and platelet count <100,000 cells/mm^3^), imaging findings on chest X-ray and computed tomography (CT) scans showing irregular patchy or flake-like pulmonary infiltrates, and clinical manifestations including cough, expectoration, fever, respiratory rate ≥30 breaths/min, and an arterial oxygen partial pressure to inspired oxygen fraction ratio (PaO_2_/FiO_2_) ≤ 250 mmHg; ② Those requiring mechanical ventilation; ③ Those identified as having a high nutritional risk based on nutritional risk screening after admission; ④ Those with stable vital signs and without communication barriers.

The exclusion criteria were as follows: ① Patients with other coexisting organic pulmonary diseases, such as tuberculosis or lung malignancy; ② Those with severe gastrointestinal dysfunction, including intestinal obstruction, perforation, or ischemia; ③ Those with severe cardiac, hepatic, or renal diseases or dysfunction; ④ Those with conditions that precluded prone positioning, such as cerebral hemorrhage or pneumothorax; ⑤ Those iwho were intolerant to enteral nutrition.

### Sample size estimation

Given the retrospective nature of this study, a post hoc power analysis was performed to evaluate whether the sample size met statistical requirements. The analysis was conducted using G*Power software (version 3.1.9.7), with a two-sided significance level (α) of 0.05. Based on the primary outcome indicators of this study, an effect size (Cohen’s d) of 0.80 was assumed. With 35 patients in the control group and 20 patients in the observation group, the calculated statistical power (1−β) was 0.80, indicating that the sample size was adequate for statistical analysis.

### Treatment methods

All patients received routine examinations, anti-infective therapyand standard internal medicine care. Antimicrobial agents were administered according to individual clinical conditions and etiological findings. Changes in patients’ clinical status and vital signs were closely monitored, including signs of dysfunction in the digestive, nervous, and circulatory systems. Physicians were promptly notified if patients developed symptoms such as pallor, dyspnea, or respiratory distress. Respiratory secretions were routinely cleared, and nebulized sputum suction using ambroxol was performed when necessary to maintain airway patency. In addition, health education was provided to patients’ family members, including information on the pathophysiology of severe pneumonia, common complications, and relevant nursing measures.

Patients in the control group received conventional mechanical ventilation combined with early enteral nutrition support. Mechanical ventilation was performed in the supine position, with an inspired oxygen concentration of 40%–50%, inspiratory pressure of 15–30 cmH_2_O, and positive end-expiratory pressure (PEEP) of 5–15 cmH_2_O. Enteral nutrition was initiated within 24 h after the initiation of mechanical ventilation. Nutritional risk screening was used to assess patients’ nutritional status, and individualized nutritional support plans were developed accordingly. A whole-protein enteral nutrition formula (trade name: Nutrison®, Nutricia Pharmaceutical Co., Ltd., National Drug Approval No. H20130888) was administered, with a caloric supply of 20–25 kcal/(kg·day). The enteral nutrition solution was warmed and maintained at approximately 37 °C and delivered via a nasogastric tube. Each infusion volume was 250 mL, with a total daily volume of 1000–1250 mL. The initial infusion rate was set at 10–20 mL/h and gradually increased based on patients’ tolerance to meet nutritional requirements. The intervention was continued for 1 week.

Patients in the observation group received prone position mechanical ventilation combined with early enteral nutrition support. Before positioning, oral, nasal, and airway secretions were thoroughly suctioned. Prone positioning was performed by four healthcare workers: one at the head of the bed to stabilize the head and secure all tubes, two positioned at the sides of the patient, and one assisting with coordination of the procedure. Under unified command, the patient was first moved laterally and then carefully turned into the prone position and placed on a hospital-made sponge mattress designed for prone positioning. A concave pillow was placed under the patient’s head, and the body was positioned comfortably and functionally. The patient’s upper limbs were positioned vertically along both sides of the body. The patency and fixation of all tubes were checked to prevent displacement. For patients with agitation, appropriate sedation and analgesia (e.g., dexmedetomidine, propofol, fentanyl, or remifentanil) were administered as needed to reduce the risk of unplanned extubation. During prone position mechanical ventilation, vibration-assisted sputum expectoration or chest percussion was routinely performed, and intermittent massage was applied to pressure-prone areas. Heart rate, blood pressure, and transcutaneous oxygen saturation were continuously monitored. If no abnormalities were observed, electrocardiographic electrodes and leads were repositioned to the patient’s back. The protocol for early enteral nutrition support in the observation group was identical to that used in the control group. The intervention was maintained for 1 week.

### Observation indicators

Changes in blood gas indicators before and after the intervention were compared, including partial pressure of oxygen (PaO_2_), partial pressure of carbon dioxide (PaCO_2_), and blood oxygen saturation (SpO_2_). The oxygenation index was calculated as the ratio of PaO_2_ to the fraction of inspired oxygen (PaO_2_/FiO_2_).

Changes in nutritional status and inflammatory indices before and after the intervention were also evaluated. Fasting venous blood samples (5 mL) were collected from all patients and centrifuged at 3,500 r/min for 10 min, after which the supernatant was obtained for biochemical analysis. Nutritional status was assessed using serum prealbumin (PAB), albumin (ALB) and haemoglobin (HGB), which were measured by a fully automatic biochemical analyzer. Inflammatory markers included C-reactive protein (CRP) and procalcitonin (PCT), which were measured by enzyme-linked immunosorbent assay (ELISA) and immunochemiluminescent assay (ICA), respectively. The HGB and CRP assay kits were purchased from Shenzhen Mindray Bio-Medical Electronics Co., Ltd.; the ALB assay kit was obtained from Beckman Coulter, Inc. (USA); the PAB assay kit was purchased from Zhejiang Qiangsheng Biotechnology Co., Ltd. (KINGSBIO); and the PCT assay kit was obtained from Abbott Laboratories (USA). All experimental procedures were strictly performed in accordance with the manufacturers' instructions.

The incidence of adverse events during the intervention was compared between the two groups. Adverse events were defined as additional injuries or complications occurring during mechanical ventilation, including ventilator-associated pneumonia, feeding intolerance, hemodynamic fluctuations, tube displacement or dislodgement, aspiration, and pressure injuries. Feeding intolerance was defined as the occurrence of vomiting, abdominal distension, diarrhea, or gastric retention.

### Statistical analysis

Statistical analyses were performed using GraphPad Prism 8.0 software (Graph Pad Inc., La Jolla, CA, USA). The Shapiro-Wilk test was used to assess the normality of measurement data. Measurement data conforming to a normal distribution were expressed as mean ± standard deviation. Paired sample t-tests were used for within-group comparisons, and independent sample t-tests were used for between-group comparisons. Measurement data conforming to a skewed distribution were statistically described using the median (Q1,Q3). The Mann-Whitney U test was used for between-group comparisons, and the Wilcoxon matched-pairs signed rank test was used for within-group comparisons. Categorical data were expressed as percentages (%) and analyzed using the *χ* ^*2*^ test or Fisher’s exact test. The test level was α = 0.05, and *P* < 0.05 was considered a statistically meaningful difference.

## Results

### Characteristics of the study population

**FIGURE 1:**
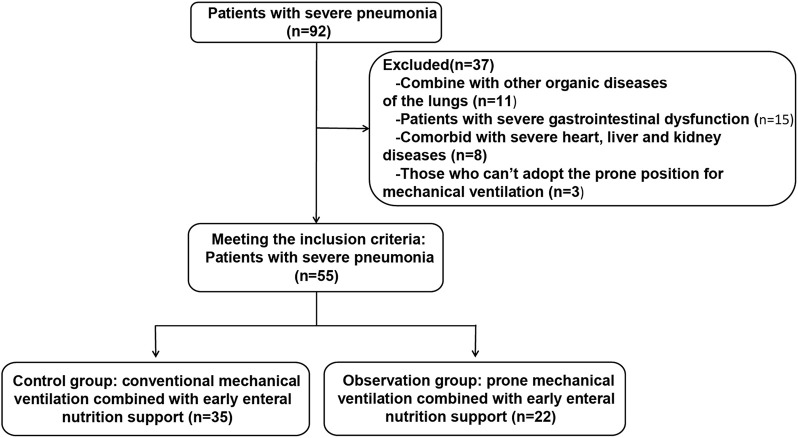
Patient enrolment and study profile.

From August 2019 to December 2023, a total of 92 patients with severe pneumonia were admitted to our hospital. Among them, 37 patients were excluded based on the exclusion criteria, and 55 patients were ultimately included in the analysis. These patients were assigned to a control group (conventional mechanical ventilation combined with early enteral nutrition support) or an observation group (prone position mechanical ventilation combined with early enteral nutrition support) according to the intervention received. The flowchart of patient enrollment and group allocation is shown in Figure 1. In the control group, there were 20 male and 15 female patients, with a mean age of 51.86 ± 4.95 years, a mean body weight of 71.61 ± 7.13 kg, and a disease duration of 10.69 ± 3.00 days. In the observation group, there were 13 male and seven female patients, with a mean age of 50.80 ± 4.70 years, a mean body weight of 72.40 ± 7.51 kg, and a disease duration of 11.30 ± 2.89 days. There were no significant differences in baseline characteristics, such as gender and age, between the two groups (*P* > 0.05), indicating good comparability. The duration of mechanical ventilation and length of hospital stay were shorter in the observation group than in the control group (*P* < 0.001) (Table 1).

**TABLE 1:** Characteristics comparison between the observation group and the control group.

Indicators	Control group (n = 35)	Observation group (n = 20)	*P*
Gender			0.775*
Male	20 (57.14%)	13 (65.00%)	
Female	15 (42.86%)	7 (35.00%)	
Age (year)	51.86 ± 4.95	50.80 ± 4.70	0.442
Weight (kg)	71.61 ± 7.13	72.40 ± 7.51	0.701
Pathogen type			0.801
Gram-positive bacteria	15 (42.86%)	7 (35.00%)	
Gram-negative bacteria	16 (45.71%)	11 (55.00%)	
Fungus	4 (11.43%)	2 (10.00%)	
Duration of disease (day)	10.69 ± 3.00	11.30 ± 2.89	0.462
Mechanical ventilation time (day)	9.17 ± 1.92	6.00 ± 1.21	<0.001
Hospital stay (day)	14.66 ± 3.24	10.55 ± 3.05	<0.001

* indicates the use of Fisher’s exact test.

### Changes in blood gas indicators before and after the intervention

Compared with pre-intervention values, PaO_2_, SpO_2_, and PaO_2_/FiO_2_ increased, while PaCO_2_ decreased in both groups after the intervention. After intervention PaO_2_, SpO_2_, and PaO_2_/FiO_2_ were higher and PaCO_2_ was lower in the observation group than in the control group (*P* < 0.05). These findings suggest that enteral nutrition support combined with prone position mechanical ventilation may contribute to improved respiratory function in patients with severe pneumonia (Table 2).

**TABLE 2:** Changes in blood gas indicators before and after the intervention in two groups of patients.

Groups	PaO_2_ (mmHg)		PaCO_2_ (mmHg)	
	Before intervention	After intervention	Before intervention	After intervention
Control group (n = 35)	57.14 ± 4.39	89.09 ± 5.32*	55.34 ± 5.08	46.91 ± 4.03*
Observation group (n = 20)	57.40 ± 4.01	95.05 ± 4.08*	55.85 ± 5.14	41.60 ± 3.56*
*P*	0.830	<0.001	0.724	<0.001

Groups	SpO_2_ (%)		PaO_2_/FiO_2_ (mmHg)	
	Before intervention	After intervention	Before intervention	After intervention
Control group (n = 35)	88.00 (83.00,92.00)	92.0 (87.00,97.00)*	189.70 ± 16.59	298.60 ± 22.64*
Observation group (n = 20)	88.00 (86.00,91.75)	95.50 (93.25,99.00)*	190.30 ± 17.82	327.60 ± 25.50*
*P*	0.771	0.012	0.850	<0.001

*P < 0.05 vs. the same group before intervention.

### Nutritional status before and after the intervention

After the intervention, PAB, ALB, and HGB increased in both groups compared with baseline, with higher levels observed in the observation group than in the control group (*P* < 0.05). However, there were no statistically significant differences in body weight between the two groups before and after the intervention (*P* > 0.05). These results indicate that enteral nutrition support combined with prone position mechanical ventilation may be beneficial for improving nutritional status in patients with severe pneumonia (Table 3).

**TABLE 3:** Comparison of the nutritional status two groups of patients before and after the intervention.

Groups	PAB (g/L)		ALB (g/L)		HGB (g/L)		Weight (kg)	
	Before intervention	After intervention	Before intervention	After intervention	Before intervention	After intervention	Before intervention	After intervention
Control group (n = 35)	172.90 ± 14.18	185.10 ± 14.54*	32.98 ± 3.67	36.14 ± 3.38*	92.51 ± 6.81	98.55 ± 7.81*	71.61 ± 7.13	73.04 ± 9.25
Observation group (n = 20)	173.20 ± 15.13	206.60 ± 23.81*	31.87 ± 3.32	39.53 ± 4.47*	93.35 ± 7.03	105.70 ± 8.15*	72.40 ± 7.51	76.12 ± 10.39
*P*	0.941	<0.001	0.270	0.003	0.668	0.002	0.701	0.262

*P < 0.05 vs. the same group before intervention.

### Changes inflammatory indicators before and after the intervention

Levels of CRP and PCT were reduced in both groups after the intervention, and Post-intervention CRP and PCT levels were lower in the observation group than in the control group (*P* < 0.05). These findings suggest that enteral nutrition support combined with prone position mechanical ventilation may help alleviate the inflammatory response in patients with severe pneumonia (Table 4).

**TABLE 4:** Comparison of inflammatory indicators before and after the intervention in both groups of patients.

Groups	CRP (mg/L)		PCT (ng/L)	
	Before intervention	After intervention	Before intervention	After intervention
Control group (n = 35)	102.70 ± 14.53	86.26 ± 8.64*	5.01 ± 1.03	3.42 ± 0.74*
Observation group (n = 20)	104.10 ± 15.25	73.30 ± 7.58*	4.80 ± 1.07	2.52 ± 0.57*
*P*	0.733	<0.001	0.473	<0.001

*P < 0.05 vs. the same group before intervention.

### Incidence of adverse reactions

In the control group, there were 9 cases of feeding intolerance, 1 case of hemodynamic fluctuations, 1 case of ventilator-associated lung injury, 2 cases of tube displacement/dislodgement, and 3 cases each of aspiration and pressure injuries; in the observation group, there were 2 cases each of the above-mentioned adverse reactions, 1 case of hemodynamic fluctuations, 0 cases of ventilator-associated lung injury, 1 case of tube displacement/dislodgement, 1 case of aspiration, and 0 cases of pressure injuries. The incidence of adverse reactions in the observation group (25.00%) was lower than that in the control group (54.29%) (*P* < 0.05), suggesting a more favorable safety profile for the combined intervention (Table 5).

**TABLE 5:** Comparison of the incidence of adverse reactions between the two groups of patients.

Groups	Feeding intolerance	Hemodynamic fluctuations	Ventilator-associated lung injury	Tube displacement/ Dislodgement	Aspiration	Pressure injuries	Total incidence
Control group (n = 35)	9 (25.71%)	1 (2.86%)	1 (2.86%)	2 (5.71%)	3 (8.57%)	3 (8.57%)	19 (54.29%)
Observation group (n = 20)	2 (10.00%)	1 (5.00%)	0 (0.00%)	1 (5.00%)	1 (5.00%)	0 (0.00%)	5 (25.00%)
*P*	0.293	>0.999	>0.999	>0.999	>0.999	0.293	0.049*

* indicates the use of Fisher’s exact test.

## Discussion

Despite ongoing advances in medical care, pneumonia remains a common infectious disease associated with substantial morbidity and mortality (Gadsby and Musher, 2022). Patients with severe pneumonia may develop profound hypoxaemia, ARDS, or even death (Vaughn et al., 2024), often necessitating mechanical ventilation. Prone positioning is one of the few interventions that has demonstrated a beneficial impact on patient outcomes, with evidence showing a significant reduction in mortality among mechanically ventilated patients with ARDS (Papazian et al., 2019). In addition, malnutrition is frequently observed in patients with severe pneumonia, highlighting the need for appropriate nutritional support. Therefore, the present study was conducted to investigate the combined efficacy of prone position mechanical ventilation and enteral nutritional support in patients with severe pneumonia.

Consistent with the findings reported by Li et al. (Li et al., 2024), prone position ventilation has been shown to improve the oxygenation index and shorten the duration of mechanical ventilation in patients undergoing cardiopulmonary bypass surgery without causing significant hemodynamic changes. In the present study, both mechanical ventilation time and length of hospital stay were shorter in the observation group than in the control group. Improvements in respiratory function were among the key findings, as patients in the observation group exhibited significant increases in PaO_2_, SpO_2_, and PaO_2_/FiO_2_, alongside reductions in PaCO_2_. These changes suggest that prone positioning may enhance alveolar recruitment and reduce ventilation–perfusion mismatch, which is consistent with previous reports (Bajon and Gauthier, 2023). Evidence has shown that prone position ventilation is typically associated with improvements in arterial blood gas parameters, primarily due to more effective ventilation–perfusion matching (Guerin et al., 2020). Moreover, it has been reported that, compared with conventional dietary support, standardized enteral nutrition can improve cardiopulmonary function—reflected by higher PaO_2_ levels and greater reductions in PaCO_2_—in patients with acute exacerbations of chronic obstructive pulmonary disease (AECOPD) complicated by respiratory failure (Zhang et al., 2021).

Improvements in nutritional status further supported the potential benefits of the combined intervention. After treatment, levels of PAB, ALB, and HGB were higher in the observation group, indicating that enteral nutrition support plays an important role in meeting metabolic demands and preventing malnutrition. By providing essential proteins, energy, and micronutrients, enteral feeding supports tissue repair, enhances immune function, and mitigates catabolic stress. These effects are particularly relevant in severe pneumonia, where systemic inflammation and metabolic disturbances often result in rapid nutritional depletion (Wells et al., 2020). Zhang et al. (Zhang et al., 2021) reported that standardized enteral nutrition improves nutritional status and immune function in patients with AECOPD complicated by respiratory failure and contributes to inflammation reduction, which is in line with the nutritional improvements observed in the present study. In addition, reductions in inflammatory markers were observed in the observation group. Levels of CRP and PCT—widely used indicators of systemic inflammation (Buerke et al., 2022; Mancino, 2023)—were lower after intervention in the observation group compared with the control group, suggesting that enteral nutrition support combined with prone position mechanical ventilation may help alleviate the inflammatory response in patients with severe pneumonia. Supporting this observation, a retrospective study reported that prone position mechanical ventilation was associated with reduced systemic inflammation, as evidenced by a progressive decrease in plasma interleukin-6 concentrations in patients receiving prone ventilation (Walter and Ricard, 2023). Enteral nutrition may further contribute to this effect by maintaining intestinal barrier integrity, reducing bacterial translocation, and modulating immune responses (Aguila et al., 2020). Nevertheless, it should be noted that CRP and PCT are acute-phase reactants whose levels are influenced by multiple factors, including infection severity, tissue injury, and physiological stress. Therefore, changes in these biomarkers cannot be solely attributed to the combined intervention. Although the observed reductions represent a favorable signal, their precise clinical significance and direct association with pulmonary inflammatory outcomes require further confirmation through future studies incorporating more specific inflammatory markers and clinically relevant endpoints.

The safety profile of the combined intervention was acceptable, as reflected by the lower incidence of adverse reactions observed in the observation group compared with the control group. The reduced occurrence of complications, such as restlessness, breathing convulsions, ventilator-associated lung injury, and gastric retention, suggests the feasibility and practicality of this integrated approach. One possible explanation for the lower rate of adverse events is that prone positioning reduces the disparity between dorsal and ventral transpulmonary pressures, leading to more uniform ventilation and thereby decreasing ventral alveolar overdistension and dorsal alveolar collapse (Petrone et al., 2021). These findings are clinically relevant, as minimizing complications is essential for improving patient outcomes and reducing healthcare burden. Existing evidence also supports the safety and importance of enteral nutrition during prone position ventilation in critically ill patients (Perez-Cruz, 2024).

In conclusion, this study demonstrates that the combination of prone position mechanical ventilation and early enteral nutrition support may reduce the incidence of adverse reactions, improve respiratory function and nutritional status, and alleviate the inflammatory response in patients with severe pneumonia. These findings provide additional clinical insights into the comprehensive management of severe pneumonia. Nevertheless, several limitations should be acknowledged. First, as a non-randomized controlled study, the results may be affected by selection bias. Second, given the nonspecific nature of inflammatory biomarkers such as CRP and PCT, changes in their levels may be influenced by multiple factors and may not accurately reflect local pulmonary inflammation. In addition, the absence of long-term follow-up precludes assessment of the impact of the combined strategy on long-term outcomes. Future studies with larger sample sizes, multicenter randomized controlled designs, longer follow-up periods, and the inclusion of more specific inflammatory and immune-related biomarkers are warranted to further validate the effectiveness and safety of this combined therapeutic approach.

## Data Availability

The raw data supporting the conclusions of this article will be made available by the authors, without undue reservation.
